# From Fixed Lineage to Dynamic States: Fibro-Adipogenic Progenitors in Skeletal Muscle Regeneration, Fibrosis, and Fibrofatty Degeneration

**DOI:** 10.3390/ijms27219774

**Published:** 2026-10-30

**Authors:** Jiaxin Sun, Fengying Ma, Ding Yang, Siyu Chen, Lingli Wang, Shuai Li, Mingjuan Gu, Wenguang Zhang

**Affiliations:** 1 College of Animal Science, Inner Mongolia Agricultural University, Hohhot 010018, China; sjx9301@163.com (J.S.); fengyingma1997@163.com (F.M.); yangding81@126.com (D.Y.); 15183400028@163.com (S.C.); 15647597596@163.com (L.W.); lishuai@email.imau.edu.cn (S.L.); 2 Key Laboratory of Animal Genetics, Breeding and Reproduction of the Inner Mongolia Autonomous Region, Inner Mongolia Agricultural University, Hohhot 010018, China

**Keywords:** fibro-adipogenic progenitors (FAPs), skeletal muscle niche, muscle regeneration, cell–cell communication, extracellular matrix remodeling

## Abstract

Skeletal muscle repair depends on coordinated interactions among muscle stem cells, immune cells, extracellular matrix, and resident stromal cells. A key stromal population, fibro-adipogenic progenitors (FAPs), has attracted considerable attention. Yet FAPs are not a fixed mesenchymal lineage. They form a plastic, heterogeneous compartment with context-dependent functional states that shift with injury stage, anatomical site, disease, and species. Understanding what drives this plasticity is essential. This review examines how biochemical, inflammatory, mechanical, and epigenetic signals shape FAP states. We synthesize evidence from multiple species, including mouse, human, bovine, porcine, and camel muscle systems. Four regulatory modules are considered: TGF-β/PDGFRα signaling and immune crosstalk, canonical and non-canonical Wnt pathways, epigenetic regulation, and extracellular matrix mechanotransduction. Through these pathways, FAPs adopt distinct functional programs. These include homeostatic maintenance, reparative support, fibrogenic activation, adipogenic differentiation, inflammatory response, and senescence. Each program differentially influences muscle stem cell function and tissue remodeling outcomes. These programs reflect context-dependent FAP states, not a uniform lineage with fixed functions. Several concepts require clarification. We emphasize that fibrogenic and adipogenic programs can be differentially regulated yet coexist within diseased muscle, and that pathological FAP states may retain context-dependent reversibility. These insights clarify the constraints on cross-species translation and inform the design of stage-matched, context-appropriate, or combinatorial FAP-directed therapeutic strategies for muscle disease.

## 1. Introduction

Skeletal muscle regeneration is a highly coordinated multicellular process, rather than an autonomous response driven solely by satellite cells [1,2]. Following injury, tissue restoration requires dynamic interactions among muscle stem cells (MuSCs), immune, extracellular matrix (ECM) components, and resident stromal populations [3,4]. Prolonged or dysregulated interactions can divert regeneration toward fibrosis, intramuscular fat accumulation, chronic inflammation, and impaired contractility across aging-related, dystrophic, metabolic, and chronic injury settings. Among the non-myogenic stromal populations implicated in these processes, fibro-adipogenic progenitors (FAPs) have emerged as critical regulators that integrate regenerative signaling and pathological tissue remodeling [5].

In skeletal muscle, FAPs were initially characterized as non-myogenic interstitial mesenchymal cells, enriched by a PDGFRα^+^ Sca-1^+^ phenotype and defined by their capacity for fibro-adipogenic differentiation [6]. This marker combination remains a widely adopted operational strategy for FAP enrichment in mouse models [7]; however, caution is warranted against treating it as a definitive lineage marker or a universal surrogate for FAP function. PDGFRα and Sca-1 provide valuable tools for distinguishing stromal populations in murine systems, whereas the biological consequences of FAP activation are strongly influenced by tissue context, activation state, anatomical niche, and interactions with neighboring cell populations. Furthermore, Sca-1 lacks a strict human ortholog, underscoring the need to separate species-specific isolation markers from conserved functional attributes of FAPs [8]. Therefore, although marker-based isolation is essential for experimental reproducibility, marker expression alone cannot determine whether a FAP population adopts reparative, fibrogenic, adipogenic, senescent, or other functional states.

During acute muscle repair, transient FAP expansion supports MuSC activation, myogenic differentiation, immune coordination, and ECM remodeling [9,10]. Persistent injury signals or unresolved inflammation favor maladaptive states: excessive matrix deposition, adipogenic differentiation, inflammatory activation, or senescence [11,12]. The same pathways may support repair or drive degeneration depending on context. Disease-specific microenvironments illustrate this principle. In aging muscle, reduced WISP1, tenascin-C, BMP3B, and IGF-I coincide with increased senescence, inflammation, and fibrosis [13,14,15,16]. In Duchenne muscular dystrophy (DMD), sustained TGF-β signaling, macrophage signals, complement–Wnt interactions, and PDGFRα pathways reinforce fibrogenic programs [10]. Chronic disuse, metabolic stress, or ischemia enhance adipogenic potential in FAP subsets, driving intramuscular fat accumulation [17]. FAP behavior reflects dynamic, context-dependent states rather than fixed lineage functions. Pathological outcomes arise from dysregulated state transitions or persistent maladaptive programs within altered biochemical and mechanical environments.

Recent reviews have summarized FAP origin, heterogeneity, and therapeutic potential [6,9,10,18]. Yet a critical question remains unanswered: why do FAPs support regeneration in some contexts but promote fibrosis, adipogenic remodeling, or chronic inflammation in others? This review addresses these divergent outcomes through the lens of dynamic, context-dependent FAP states rather than a fixed lineage with predetermined functions. We synthesize evidence on how inflammatory signals, extracellular matrix mechanics, transcriptional regulation, and epigenetic memory jointly shape FAP states. Particular attention is given to interactions among these regulatory inputs and how they sustain pathological programs. By comparing findings across species and disease stages, we clarify the distinction between FAP identity and functional state. We also discuss the conditions that permit reversibility versus those that favor pathological persistence. These insights may inform rational intervention strategies. Therapeutic approaches can be matched to disease stage, tailored to local context, and designed as combinatorial regimens targeting multiple regulatory axes. Such FAP-directed interventions hold promise for improving outcomes in muscle injury, degenerative disease, and metabolic disorders.

## 2. Literature Search and Selection Strategy

This critical narrative review was based on a systematic search of PubMed, Web of Science, and Scopus (Google Scholar used only for manual backward citation chasing) from inception to June 2026. Search terms combined FAP identity descriptors (“fibro-adipogenic progenitors”, “FAPs”, “non-myogenic mesenchymal progenitors”) with skeletal-muscle topic terms (heterogeneity, regulation, aging, disease, interventions); identity terms were linked by OR and intersected with topic terms by AND. Retrieval targeted FAP biology broadly, not merely nomenclature variants.

Eligible studies investigated skeletal-muscle-resident or clearly FAP-enriched populations and addressed identity, localization, lineage potential, functional heterogeneity, niche interactions, regulation, aging/disease states, or interventions; in vivo, ex vivo, and in vitro designs (lineage tracing, sc/snRNA-seq, spatial transcriptomics, perturbation assays) were included. Mouse, human, and livestock models were retained for cross-species comparison. Reviews supplied context only. Exclusions comprised non-muscle stromal work, abstracts/editorials, duplicates, and studies where FAP findings were indistinguishable from other stromal cells.

Evidence strength was appraised qualitatively by FAP-identification specificity, design rigor, directness of functional evidence, and cross-study consistency; genetic/lineage-tracing evidence outranked marker or transcriptomic associations, and in vitro findings were not equated with in vivo mechanisms without corroboration. Conflicting results were retained and compared systematically, with divergence attributed to species, anatomical site, isolation protocol, marker thresholds, age, disease stage, or culture conditions; unresolved discrepancies were acknowledged explicitly. Two authors screened independently; disagreements were resolved by a third. No quantitative pooling was performed given study heterogeneity.

## 3. Defining FAP Identity, Heterogeneity, and Cellular States

FAP identity depends on more than a surface-marker combination. Anatomical position, differentiation capacity, and transcriptional state also vary with species, developmental stage, muscle, and disease. Early studies defined FAPs as PDGFRα+ mesenchymal progenitors with fibro-adipogenic potential, but later work showed that this label encompasses several functionally distinct populations. A reliable description therefore combines flow cytometry, lineage tracing, differentiation assays, single-cell transcriptomics, and spatial mapping (Table 1). This section first reviews practical identification methods and then considers how single-cell and comparative studies have shifted the view of FAPs from a uniform progenitor pool to a heterogeneous stromal compartment.

### 3.1. Phenotypic Identification and Functional Annotation of FAPs

Skeletal muscle regeneration depends on several resident stem and progenitor populations, including FAPs, but isolating FAPs reproducibly remains difficult [39]. Unlike muscle satellite cells, FAPs lack a single exclusive surface antigen. Researchers therefore use combinations of positive and negative markers, and the resulting population reflects both the gating strategy and the biological context [19].

Fluorescence-activated cell sorting (FACS) is useful because it measures protein expression in intact tissue and allows exclusion of hematopoietic, endothelial, and myogenic contaminants. The CD31^−^/CD45^−^/Integrin-α7^−^/Sca-1^+^ panel established by Rossi and colleagues became a common starting point for mechanistic studies [19,20]. Later protocols added PDGFRα enrichment [21], VCAM1 exclusion [35], or age-specific PDGFRβ-negative gates [40]. These refinements trade yield for purity, and reported FAP frequencies of 2.6% to 17.5% may therefore reflect differences in gating as well as biological variation.

Isolation must also follow the disease and developmental setting. Dystrophic models may require expanded panels such as Ter119^−^/CD34^+^/Sca-1^+^ to capture activated subsets [39], whereas developmental tracing has identified Osr1 in embryonic FAP-like mesenchyme [27]. Human samples show a further layer of divergence. Human and murine cells share a CD31^−^/CD45^−^/CD56^−^ negative profile, but human FAPs are enriched by CD34^+^ and can be further resolved with CD201 and CD73 [24]; viability exclusion is important for robust recovery [22,23]. These methods made FAPs experimentally accessible, yet marker-based gating captures a time-specific sample of a heterogeneous stromal compartment. Single-cell and spatial approaches are needed to connect marker profiles with local signals and function.

### 3.2. Single-Cell and Cross-Species Resolution of FAP Heterogeneity

Single-cell and single-nucleus studies extend immunophenotypic identification by resolving transcriptional variation within the FAP compartment. LUM- and FBN1-associated populations occur in human and mouse muscle, with shared PDGFRA/CD34 expression and ECM-related programs [26]. Their correspondence is partial: TEK and collagen-expression patterns differ between the datasets. In particular, COL14A1 enrichment in human FBN1-associated cells suggests a perimysial relationship, whereas COL4/COL15-associated expression in LUM cells suggests an endomysial relationship; these transcriptional observations do not themselves constitute spatial or lineage validation [26].

Cross-species comparison of adipogenic competence illustrates why state annotation needs functional testing. Fitzgerald et al. identified MME^+^ FAPs with greater adipogenic potential than MME^−^ cells in both species [25]. The difference was evident under minimal induction in mouse cells, whereas in human cells it persisted under full adipogenic stimulation, with very few MME^−^ cells differentiating. Thus, adipogenic competence may be more restricted to particular human subsets under the tested conditions. This does not establish uniformly lower adipogenic potential for human FAPs as a whole: subset composition, muscle source and induction conditions must be considered. The same study combined human differentiation assays and tissue observations with mouse transplantation experiments; these evidence types should not be treated as equivalent to endogenous lineage tracing in humans [25].

Livestock studies extend this comparison at different levels of validation. In cattle, COL4A1/CFD-enriched adipogenic and COL1A1/POSTN-enriched fibrogenic FAP programs were associated with endomysial and perimysial ECM patterns, respectively [25]. Bovine culture perturbations supported CFD-associated adipogenic regulation, whereas Postn lineage-tracing validation in that study was performed in mice. Porcine atlases report LPL-associated cells in newborn muscle and COL3A1-, PDE4D/PDE7B- and EBF1-associated FAP states at later growth stages [29,30]. Spatial and single-nucleus data further describe porcine stromal heterogeneity, but trajectory inference and functional experiments in preadipocytes do not establish the fate of every atlas-defined FAP subset [31,32]. The reported MME^+^ camel population remains a transcriptomically annotated candidate associated with developmental differences in fat deposition, without direct subset-specific differentiation or lineage-tracing validation in that study [33].

Disease and age provide additional context. Human DMD FAPs show CHI3L1, EFEMP1, MFAP5 and TGFBR2-associated changes after isolation and short-term culture [36], whereas Cxcl5/Ccl7-rich activated states were identified in a dystrophic mouse study [34]. Human CD90^+^ FAPs exhibit enhanced ECM-related properties in type 2 diabetes [22]. The human ageing atlas reports a reduced MME^+^ fraction and an increased fibroblast-like fraction, rather than a general expansion of MME^+^ cells [25]. In CLTI, VCAM1^+^ FAPs show increased adipogenic competence in mouse culture assays, while human data provide transcriptomic support for the associated state and regulatory genes [38]. These examples caution against assigning an invariant function to the same surface marker across injury settings. Together, the available studies support conservation of a PDGFRα-associated interstitial stromal compartment and partial conservation of ECM and adipogenic programs. Exact isolation panels, subset proportions and differentiation responses remain species- and context-dependent (Table 1). Cross-species transcriptomic resemblance therefore motivates functional comparison; it does not establish identical lineage relationships, response thresholds or therapeutic effects.

## 4. Regulatory Axes Governing FAP Function in the Skeletal Muscle Niche

**Figure 1:**
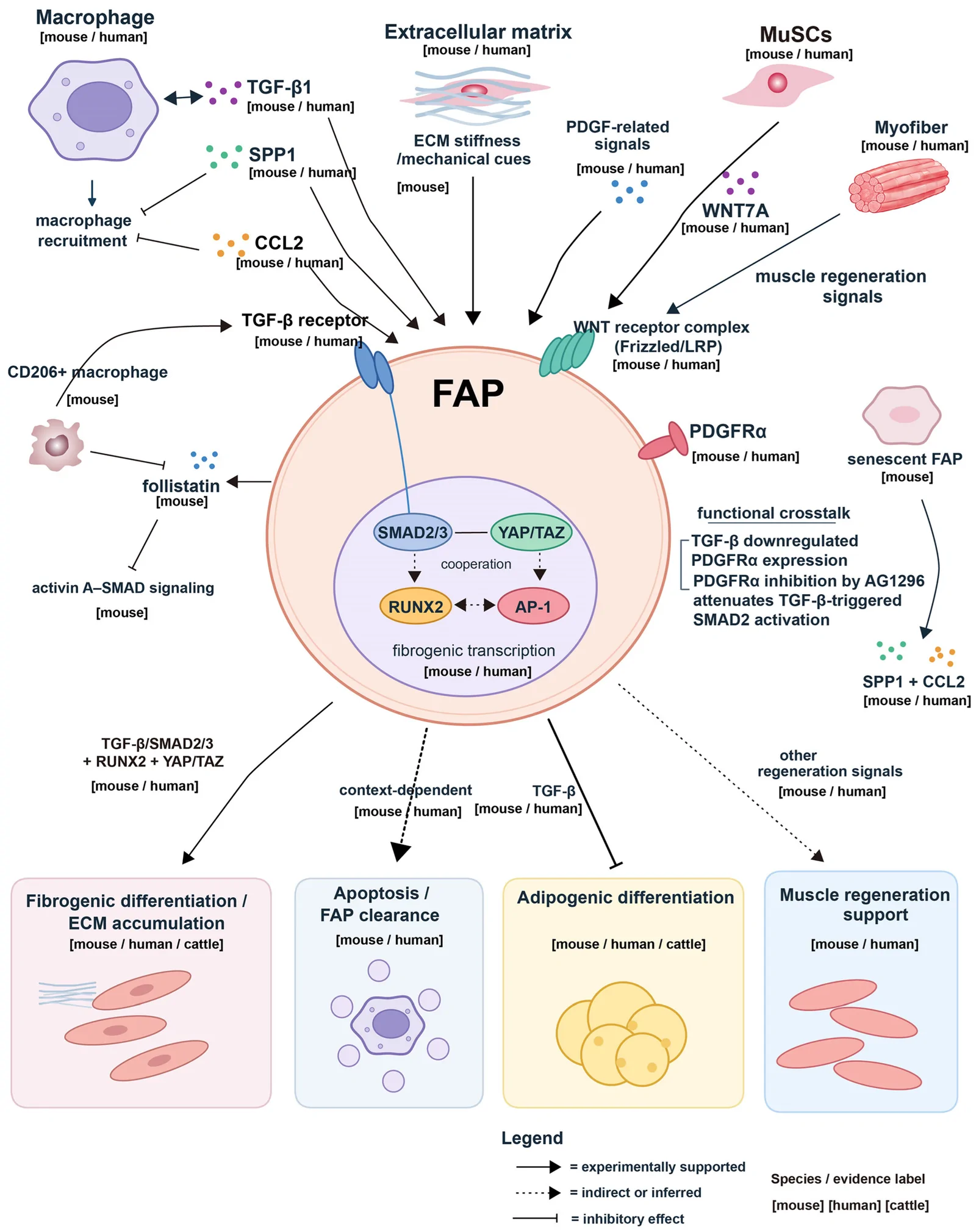
Microenvironmental regulation of dynamic FAP states and tissue outcomes. Signals from macrophages, muscle stem cells, myofibers, and extracellular matrix integrate with intracellular regulatory programs in fibro-adipogenic progenitors (FAPs). TGF-β/PDGFRα crosstalk, Wnt signaling, epigenetic regulation, and mechanosensing influence FAP activation, secretory function, differentiation, and survival. The central FAP represents a heterogeneous stromal compartment, not a single cell performing every depicted response. Outputs summarize functional programs and tissue outcomes, not fixed lineages or obligatory differentiation sequences. Fibrotic matrix accumulation and fatty infiltration can coexist within diseased muscle without establishing dual differentiation of individual FAPs. TGF-β effects on fibrogenesis, adipogenesis, and survival depend on model and cellular context (Section 4.1). Solid connectors denote experimentally supported effects; dashed connectors denote inferred relationships. Arrowheads indicate promotion or directional communication; T-bars indicate inhibition. Connectors do not necessarily represent direct molecular binding. Species and model labels identify the context of supporting evidence and do not imply universal conservation. ECM, extracellular matrix; MuSCs, muscle stem cells.

FAP behavior in skeletal muscle is not determined by intrinsic programming alone, but by integrating biochemical, epigenetic, and mechanical signals from neighboring cells and ECM. Homeostatic cues maintain FAP quiescence while supporting MuSCs, macrophages, myofibers, and ECM turnover. Injury or disease rewires these inputs, driving FAPs toward transient pro-regenerative activation or persistent fibrogenic and adipogenic pathology [41]. Four regulatory axes govern this plasticity: TGF-β–macrophage–FAP crosstalk linking inflammation to fibrosis; Wnt/β-catenin as a context-dependent switch restraining adipogenesis or promoting fibrosis; epigenetic mechanisms (miRNAs, DNA methylation, chromatin remodeling) stabilizing fate decisions; and ECM mechanics reinforcing or reversing lineage bias (Figure 1). These pathways collectively explain FAP transitions between niche-supportive and pathological states.

### 4.1. TGF-β–Macrophage–FAP Crosstalk in Fibrosis and Regenerative Failure

TGF-β promotes fibrogenic differentiation of FAPs through SMAD-dependent signaling. Concurrently, it suppresses adipogenic differentiation in experimental settings [42,43]. These observations reveal context-dependent regulation of fibrogenic and adipogenic programs. Importantly, they do not support an obligatory fate switch. At the tissue level, fibrotic matrix accumulation and fatty infiltration frequently coexist in diseased muscle [44,45]. However, this coexistence does not establish simultaneous activation of both programs within individual FAPs. Consequently, the outcomes of TGF-β inhibition cannot be extrapolated solely from its anti-adipogenic effects in culture. For instance, in a mouse model of massive rotator cuff tear, SB431542 treatment reduced both fibrosis and fatty infiltration. Notably, it also increased FAP apoptosis [46]. These findings underscore the necessity of evaluating TGF-β-directed interventions for their dual effects on FAP differentiation and survival.

During acute injury, FAPs transiently expand to support regeneration. Subsequently, they undergo apoptosis during resolution. FAP-specific AMPKα1 deletion disrupts this process. It elevates active TGF-β1 levels, impairs apoptosis, and promotes fibrogenic differentiation [47]. This cascade links defective FAP clearance to persistent matrix deposition. RUNX2, a TGF-β-responsive transcription factor, further drives fibrogenic gene expression in FAPs. Pharmacological RUNX2 inhibition with CADD522 reduced fibrosis in dystrophic mice [11]. Nevertheless, potential effects on bone homeostasis warrant consideration, given RUNX2’s essential role in osteoblast differentiation. In parallel, TGF-β/SMAD3 recruits the mechanosensitive co-transcriptional regulators YAP and TAZ. This recruitment amplifies fibrogenic transcription [42]. Additionally, TGF-β–PDGFRα crosstalk involves reciprocal modulation. TGF-β downregulates PDGFRα expression, whereas PDGFRα inhibition attenuates TGF-β-induced SMAD2 activation [43]. These interactions indicate functional crosstalk rather than uniform synergy.

Macrophage-derived TGF-β1 regulates FAP behavior after injury. However, macrophage–FAP communication extends beyond this single ligand. Depletion of CD206^+^ M2-like macrophages enhanced FAP-derived follistatin secretion and accelerated regeneration [48]. Similarly, TGF-β1 deletion in CD206^+^ macrophages improved muscle regeneration. Conversely, FAP-specific follistatin deletion in obese mice activated myostatin/activin A–SMAD signaling. This activation reduced muscle mass and impaired exercise performance [49]. SPP1 (osteopontin) represents another communication axis. Senescent FAPs secreted SPP1 and CCL2, and neutralization of either factor reduced macrophage recruitment in vitro [50]. Moreover, in dystrophic muscle, macrophage-specific Spp1 ablation reduced two PDGFRα^+^ adipogenic stromal populations (Lifr^+^ and Procr^+^). This reduction decreased intramuscular fat and improved diaphragm function [45].

FAP responses also vary with cellular state and microenvironment. Single-cell profiling of D2-mdx mice identified enrichment of a fibrosis-associated CD55^+^ FAP cluster [51]. In inflammatory myopathy, TGF-β and EGF exposure altered AP-1 element accessibility in human FAPs [52]. Furthermore, breed comparisons in cattle revealed adipogenesis-enriched FAP states in Wagyu. In contrast, Qinchuan cattle exhibited enrichment of ECM- and TGF-β/TNFα/NF-κB-related programs [53]. Additionally, irradiation induced persistent fibrotic and senescent FAP signatures with inferred monocyte/macrophage-to-FAP TGF-β signaling [54]. Collectively, these findings demonstrate context-dependent FAP regulation across disease states and genetic backgrounds.

### 4.2. Canonical and Non-Canonical Wnt Signaling in FAP State Regulation and Niche Communication

Transcriptomic analyses have revealed that FAPs express multiple Wnt ligands, including Wnt1, Wnt2, Wnt4, Wnt5a, Wnt7a, Wnt10a, and Wnt16, alongside Frizzled receptors in mouse, bovine, and human skeletal muscle [55,56]. These observations position FAPs as both sources and targets of Wnt signals. However, they do not prove that FAPs form a self-contained autocrine circuit.

Canonical Wnt/β-catenin signaling provides the clearest example of a FAP-autonomous effect on adipogenesis. The most direct evidence comes from studies of the GSK3/β-catenin branch: in isolated murine FAPs, pharmacological inhibition of GSK3 with LY2090314 or CHIR99021 stabilizes β-catenin and suppresses PPARγ/C/EBPα-driven adipogenesis [56], as it occurs in purified FAP cultures without other cell types. Bovine FAP cultures display a comparable response: GSK3 inhibition reduces adipogenic gene expression, whereas XAV-939-mediated blockade of β-catenin signaling promotes lipid accumulation [55], again consistent with direct action on FAPs. Notably, although WNT5A is conventionally classified as a non-canonical ligand, its anti-adipogenic effect in this context was traced to GSK3/β-catenin [56], underscoring that pathway assignment should be defined by downstream mechanisms rather than ligand nomenclature alone. Direct anti-adipogenic activity has also been recapitulated in human FAPs cultured in three-dimensional PEG-fibrinogen matrices [57], supporting cross-species conservation of this canonical mechanism. Importantly, in these culture systems, suppression of adipogenesis reflects a FAP-autonomous response, whereas the enhanced myogenesis observed after GSK3 inhibition is an indirect consequence: GSK3 inhibition increases FAP secretion of follistatin, which in turn promotes MuSC differentiation. Thus, the myogenic effect is paracrine, not cell-autonomous to FAPs.

Beyond this canonical branch, non-canonical Wnt signaling adds a further layer of regulation, though the evidence for FAP-intrinsic action is less complete. WNT7A inhibits FAP adipogenic differentiation through the alternative Wnt–Rho–YAP/TAZ axis [58], demonstrating functional convergence with the canonical branch despite divergent downstream effectors. However, whether this axis operates cell-autonomously in FAPs has not been definitively established; current evidence is based on cultured FAPs but would benefit from genetic loss-of-function studies in isolated cells. Importantly, the net effect of Wnt signaling on FAP fate is ligand-, receptor-, and context-dependent. Wnt3a, for instance, has been reported to promote adipogenesis in certain mesenchymal contexts, and the specific Frizzled receptors mediating anti-adipogenic responses in FAPs remain unidentified.

In disease models, Wnt signaling can support fibrogenic responses, whereas reduced pathway activity is associated with adipogenic responses in other settings. These findings depend on the ligand, cell state, and local environment. They do not establish a universal high-Wnt/low-Wnt fate switch. In DMD, work from Florio and co-workers established that FAPs and macrophages cooperate to trigger dysregulated Wnt activity: each cell population secretes unique C1 complement subunits that together fuel pathological Wnt signaling. Targeted pharmacological inhibition of C1r/s reversed this cascade, repressing Wnt transduction, ablating the fibrogenic phenotype of FAPs, and ameliorating dystrophic disease features in mdx mouse models [59]. Wnt fibrogenic axis was extended to muscle aging by Kanugovi and colleagues, who showed that aged macrophages upregulate C1q while aged FAPs elevate C1r and C1s; C1q exposure activates Wnt-driven collagen expression and fibrogenic differentiation in FAPs, and genetic ablation of C1s or systemic complement inhibition suppressed Wnt-dependent fibrosis in both aged and dystrophic mice [60]. Importantly, these studies demonstrate that the pathological Wnt signal is amplified by macrophage–FAP crosstalk; the FAP response is cell-intrinsic, but the initiating signal is microenvironment-derived. Conversely, in dysferlin-deficient muscle, Wnt signaling is markedly reduced in FAPs and correlates with enhanced adipogenic differentiation [25]. Whether this reduction is FAP-autonomous or secondary to the inflammatory/damage milieu remains to be determined. Human MME^+^ FAPs, the dominant adipogenic population, exhibit low Wnt pathway activity coupled with resistance to Wnt activators [25,61]; this resistance is observed in isolated human FAPs and may reflect an intrinsic property, but the underlying mechanisms are not fully defined. A systemic dimension was revealed by the discovery that aged small intestine-derived extracellular vesicles deliver miR-214-3p to muscle, targeting β-catenin to suppress Wnt signaling and promote FAP adipogenesis [62], systemic influence on FAPs. Additionally, MST1/2 kinases modulate FAP differentiation responses through YAP and Wnt/β-catenin activity [63], and SFRP1, an endogenous Wnt antagonist, was identified as a node through which ginsenoside Rb1 suppresses both fibrosis and fat deposition in rotator cuff tears [63,64]. Whether these manipulations act directly on FAPs or via other cells is not yet clear. Future studies should combine FAP-specific genetic perturbation with ligand- and receptor-resolved assays. This would separate autonomous Wnt responses from indirect effects driven by MuSCs, macrophages, myofibers, or systemic signals.

### 4.3. Epigenetic Regulation of FAP States and Niche Communication

Epigenetic regulation does not act as a set of isolated switches; it tunes the response of PDGFRα+ FAPs to injury signals and thereby biases the balance between regenerative support and fibro-adipogenic differentiation. Intracellular miRNAs provide relatively rapid and reversible control: miR-206 restrains adipogenesis by repressing RUNX1, whereas miR-22-3p limits the KLF6/MMP-14 axis and favors fibroblast output during repair [65,66]. miR-378 illustrates the context dependence of this layer: its loss increases adipogenic competence in vitro but does not aggravate fat deposition after acute glycerol injury in vivo [67]. These effects intersect with Wnt and TGF-β signaling, whose outputs depend on the cellular and experimental context. PDGFRα identifies an enriched stromal compartment but does not specify a fixed functional state. Thus, miRNA changes can transiently shift pathway sensitivity without necessarily fixing cell identity.

Extracellular-vesicle (EV) miRNAs extend this regulation across the niche and provide temporal feedback between FAPs, MuSCs, and macrophages. Early FAP EVs enriched in miR-127-3p promote MuSC myogenesis by suppressing S1pr3, whereas late MuSC EVs carrying miR-206-3p and miR-27a/b-3p repress FAP adipogenesis [68]. FAP exosomes enriched in miR-125b-5p at day 10 promote pro-regenerative macrophage polarization through PTPN1/ERK inhibition [69]. EV cargo therefore changes with the repair phase and can modulate TGF-β, Wnt, and YAP/TAZ activity indirectly through recipient-cell signaling. The reversibility of cargo loading and uptake is consistent with a short-lived regulatory layer; persistent inflammatory or mechanical cues, however, may repeatedly impose the same EV profile and prolong a pathological niche.

DNA methylation and chromatin organization can sustain transcriptional responses and differentiation biases. C/EBPβ together with TET3 removes methylation at PPARγ and C/EBPα regulatory regions, generating 5-hydroxymethylcytosine and enabling adipogenic transcription [70]. PRDM16–G9a/GLP complexes enrich H3K9me2/3 at the nuclear periphery, repress myogenic genes, and preserve fibro-adipogenic identity; disrupting this anchoring restores myogenic potential and reduces fibrofatty degeneration [71]. These mechanisms likely set the threshold at which PDGFRα+ FAPs remain responsive to Wnt-mediated adipogenic restraint or become dominated by TGF-β and YAP/TAZ-dependent fibrogenic programs. Unlike EV cargo, methylation and lamina-associated chromatin can persist after the initiating stimulus, although evidence remains strongest in cultured cells and the extent of their reversibility in vivo is unresolved.

Disease states can therefore convert repeated, initially transient signals into a self-maintaining FAP phenotype. In DMD, TGF-β1-induced miR-214-3p represses FGFR1 and weakens anti-fibrotic FGF2 signaling, while PRDM16-associated chromatin anchoring further limits myogenic recovery [72]. In disuse atrophy, IL-1β alters FAP EV cargo toward senescence-associated miRNAs (miR-let-7c, miR-let-7b, miR-181a, and miR-124), linking inflammatory signaling to muscle wasting [73]. Matrix stiffening and YAP/TAZ activation may reinforce this state by sustaining TGF-β responsiveness and chromatin accessibility. The resulting fate is best viewed as a continuum: EV and miRNA fluctuations rapidly adjust pathway output, whereas repeated TGF-β/inflammatory or mechanical exposure can consolidate methylation and chromatin changes that stabilize fibrogenic or adipogenic FAP states. Longitudinal single-cell methylation, chromatin, and EV measurements are needed to determine when this transition becomes reversible or fixed.

### 4.4. Extracellular Matrix Mechanics as Cues for FAP State Regulation

Matrix mechanics should be interpreted against tissue-level reference values. Adipose tissue is approximately 2 kPa, normal skeletal muscle is commonly reported at about 7–13 kPa (approximately 8 kPa in the muscle-bundle measurements discussed by Loomis et al.), and fibrotic muscle approaches or exceeds 20 kPa [74]. These values describe tissues, whereas collagen gels and hydrogels provide controlled experimental substrates. In the key FAP study, collagen-coated polyacrylamide substrates spanned 0.2–50 kPa. Nuclear YAP increased with substrate stiffness, and αSMA-defined myofibroblast activation differed significantly between 8 and 25 kPa. Verteporfin (0.5 μM) reduced nuclear YAP and attenuated αSMA activation on 25-kPa gels and rigid plastic, supporting a stiffness–YAP–myofibroblast axis [74]. The result should therefore be read as a threshold-like response within an experimental range, rather than as evidence that any stiff matrix inevitably produces the same FAP state.

The same study shows that mechanics and architecture are separable cues. Collagen gels used to vary concentration had moduli of only 0.1–0.4 kPa, below the physiological muscle range; increasing collagen concentration increased nuclear YAP but did not consistently increase proliferation or αSMA activation. Crosslinking, fibril diameter and alignment altered FAP responses without simply tracking bulk stiffness: crosslinking and larger fibrils reduced myofibroblast activation, alignment mainly reoriented nuclei, and fibril-size effects were not explained by YAP localization [74]. In bovine FAP cultures, increasing substrate stiffness reduced lipid accumulation across ECM coatings, but the assay does not establish that adipogenesis follows the same stiffness threshold measured for murine myofibroblast activation [75]. Thus, stiffness, deformability, fibril architecture and ligand presentation should be reported separately.

These findings place FAP mechanosensing within a dynamic niche. LPA–LPA1–YAP signaling increases migration of dystrophic FAPs and is reversible with LPA1 or YAP inhibition [76]. In aged muscle recovering from disuse, mechanotherapy restores FAP/stromal ECM-remodeling programs and collagen turnover [77]. Patient-derived FAPs from DMD, OPMD and IBM secrete collagen VI, and COL6A silencing improves myogenic fusion, showing that FAPs also remodel the mechanical niche they sense [78]. Biomimetic gelatin/alginate hydrogels designed near native muscle stiffness (~12 kPa) and with viscoelastic stress relaxation support FAP viability and pro-myogenic paracrine activity while limiting inflammatory polarization [79]. The translational implication is that softening alone may be insufficient: persistent TGF-β, collagen architecture and viscoelasticity can maintain pathological states even when bulk modulus is normalized.

## 5. Context-Dependent FAP States in Multicellular Repair and Disease Networks

**Figure 2:**
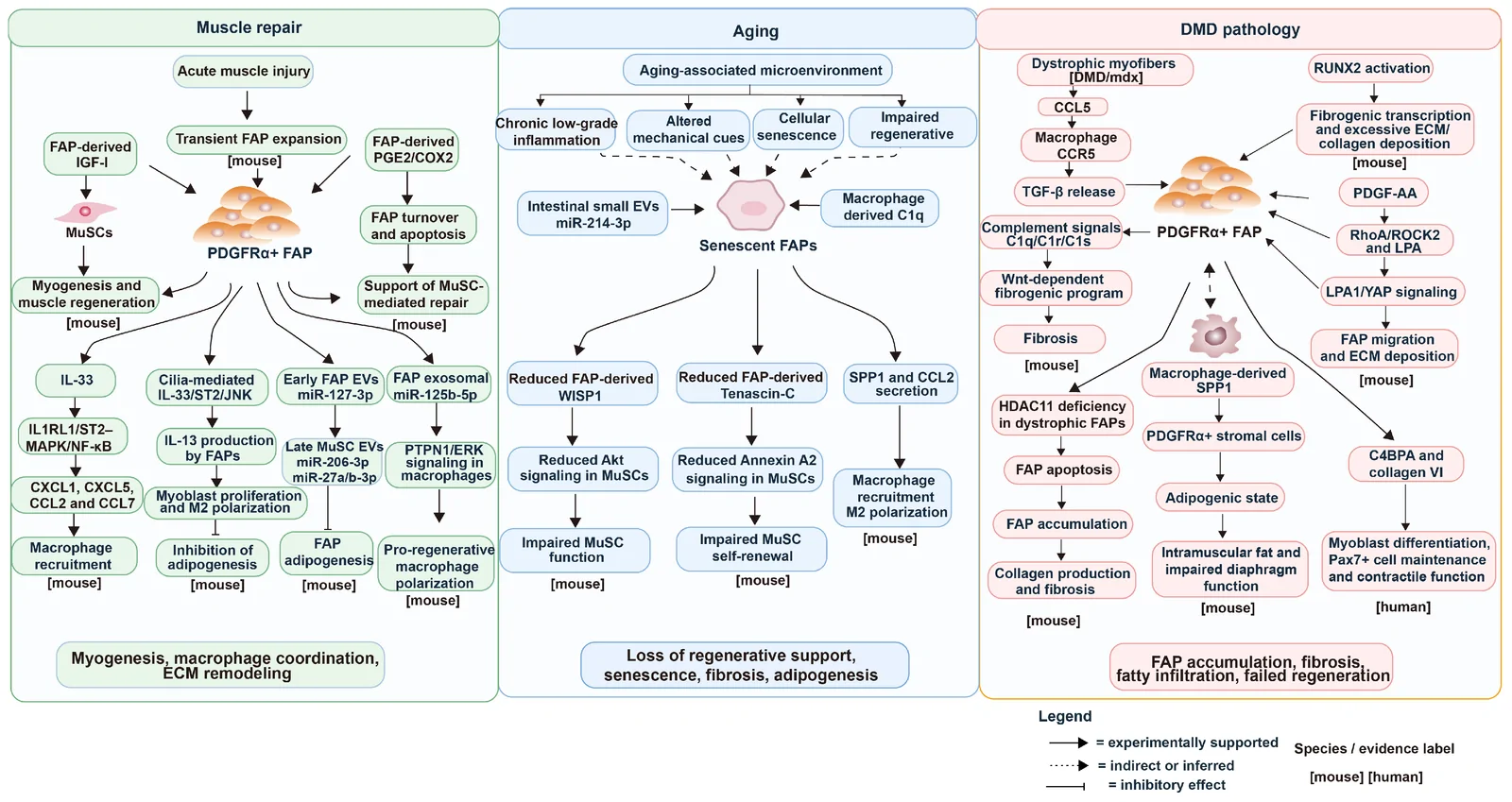
Context-dependent FAP states in muscle repair, aging, and Duchenne muscular dystrophy. The muscle-repair, aging, and DMD panels compare distinct biological settings, not successive stages of one trajectory. During repair, transient FAP activation supports muscle stem cells, immune coordination, and matrix remodeling; resolution requires FAP clearance and abundance control. Aging-associated FAP programs combine reduced regenerative support with altered secretory and inflammatory activity. In dystrophic muscle, persistent injury and niche signals drive fibrogenic, adipogenic, and regeneration-inhibiting FAP responses. Each FAP symbol represents context-dependent populations, not a uniform program shared by all cells. Fibrogenic and adipogenic outcomes can coexist within muscle without reflecting dual differentiation of individual FAPs. Intervention-associated changes may reflect altered cell state, proliferation, survival, or population composition rather than reversal of individual FAP states. Solid connectors denote experimentally supported effects; dashed connectors denote inferred relationships. Arrowheads indicate promotion or directional communication; T-bars indicate inhibition. Experimentally supported effects need not represent direct molecular interactions. Model labels distinguish animal experiments, human tissue observations, and cultured-cell findings. DMD, Duchenne muscular dystrophy; ECM, extracellular matrix; EVs, extracellular vesicles; FAPs, fibro-adipogenic progenitors; MuSCs, muscle stem cells; SASP, senescence-associated secretory phenotype.

FAP-centered multicellular networks coordinate skeletal muscle repair across physiological and pathological contexts. Rather than functioning as isolated mesenchymal progenitors, FAPs integrate signals from MuSCs, macrophages, myofibers, vascular cells, ECM, and bone-associated stromal compartments to regulate regeneration, matrix remodeling, inflammation resolution, adipogenic restraint, and osteogenic plasticity. During acute injury, this network operates transiently to support MuSC activation, immune coordination, and tissue reconstruction. In aging and muscular dystrophy, however, the same communication pathways become chronically dysregulated, generating senescent, fibrogenic, adipogenic, or disease-specific pathogenic FAP states (Figure 2). This section examines how FAP-mediated intercellular communication supports muscle repair, how it deteriorates with aging, and how subtype-specific FAP networks drive muscular dystrophy progression.

### 5.1. FAP-Centered Networks in Muscle Repair, Niche Remodeling, and Osteogenic Plasticity

FAPs are increasingly recognized as active stromal organizers of the post-injury muscle niche rather than passive fibrotic precursors. Their regenerative role depends on both intrinsic and paracrine mechanisms. FAP-specific IGF-I deletion impairs regeneration, reduces Pax7+ and MyoD+ cells, suppresses FAP proliferation, and increases inflammatory macrophages, indicating that FAP-derived IGF-I sustains both FAP fitness and MuSC-mediated repair [80]. Similarly, FAP-derived PGE2 functions as an autocrine regulator of FAP apoptosis and dynamics while maintaining the MuSC pool and promoting post-injury proliferation; FAP-specific Cox2 ablation increases FAP accumulation and fibrosis, whereas PGE2 supplementation improves regeneration and dystrophic function [81]. Thus, FAPs possess intrinsic programs that actively support repair, though these remain niche-dependent.

Early FAP activation is tightly shaped by inflammatory and myogenic cues. Single-cell studies identified an IL1RL1/ST2+ FAP state secreting CXCL1, CXCL5, CCL2, and CCL7 to recruit macrophages; autocrine IL-33 activates this program via IL1RL1-MAPK/NF-κB, and Il1rl1 deficiency impairs recruitment and regeneration [82]. In parallel, cilia-mediated IL-33/ST2/JNK signaling enhances FAP-derived IL-13, promoting myoblast proliferation and M2 polarization while limiting adipogenesis [83]. EV-mediated communication provides temporal feedback: early FAP-derived miRNAs (miR-127-3p) promote myogenesis, whereas late MuSC-derived EVs (miR-206-3p, miR-27a/b-3p) suppress FAP adipogenesis and limit IMAT [68]. FAP exosomal miR-125b-5p further induces pro-regenerative macrophage polarization via PTPN1/ERK [69]. FAPs thus coordinate repair through reciprocal communication with MuSCs and immune cells.

Whether FAPs are intrinsically pro-regenerative or mainly macrophage-instructed remains unresolved. Osr1-deficient FAPs acquire fibrogenic identity, alter matrix and cytokine secretion, and impair MuSC expansion [84]; Gli1-deficient FAPs show aberrant expansion and enhanced adipogenic potential [85]. Conversely, depletion of CD206+ M2 macrophages or macrophage TGF-β1 deletion activates FAP-derived follistatin and accelerates myogenesis, while FAP-specific follistatin loss delays repair [48]. Thus, FAP output depends on macrophage-FAP signaling timing and composition.

When this network fails to resolve, FAPs drive maladaptive remodeling. In DMD, OPMD, and IBM, FAPs are major sources of collagen VI and laminin γ1; patient-derived FAPs induce collagen deposition, myofiber atrophy, and Pax7+ depletion, while COL6 silencing restores fusion [78]. In volumetric muscle loss, β1-integrin-high FAPs secrete TGF-β1 and TIMP1, exerting pro-fibrotic and anti-myogenic effects [86]. In fatty infiltration, MME+ FAPs represent a dominant adipogenic subset with low WNT activity and reduced responsiveness to anti-adipogenic cues [25]. Pathological remodeling thus reflects persistence of disease-specific FAP states, not mere expansion.

FAP plasticity also links muscle repair to bone remodeling. Prg4+ muscle-resident FAPs migrate into fracture gaps and contribute to chondrocytes, osteoblasts, osteocytes, and periosteal progenitors; their ablation impairs fracture repair [87]. In neurogenic heterotopic ossification, M2 macrophage-derived osteopontin promotes RUNX2-dependent osteogenic differentiation via p38-MAPK [88], and in FOP and trauma models, PPARγ activation redirects FAPs from ectopic bone toward adipose [89]. FAP osteogenic potential thus reflects context-dependent plasticity, not a universal program. Overall, FAPs coordinate regeneration through IGF-I, PGE2, IL-33/ST2, IL-13, follistatin, EV-miRNAs, chemokines, and ECM remodeling. Future therapies should restore the temporal balance of FAP-centered communication rather than broadly eliminate FAPs or block single pathways.

### 5.2. Aging-Associated Disruption of FAP-Mediated Niche Coordination

In young skeletal muscle, FAP activation is transient and spatially coordinated with MuSCs, macrophages, and ECM remodeling, thereby supporting efficient myogenesis. With aging, however, this regulatory logic is progressively disrupted. Aged FAPs often expand numerically but lose regenerative quality, showing reduced pro-myogenic signaling, impaired ECM turnover, increased senescence markers, and enhanced fibrogenic or adipogenic bias. This shift directly compromises MuSC function: loss of FAP-derived WISP1 weakens Akt signaling in MuSCs, whereas reduced Tenascin-C limits Annexin A2-dependent MuSC self-renewal and repair. Consistently, FAP-specific Igf1 deletion reduces Pax7+ and MyoD+ cells, suppresses FAP proliferation and protein synthesis, perturbs ECM programs, and induces premature senescence-like features [80]. In addition, FAPs interact with MuSCs via a Tenascin-C (TnC)-dependent signaling axis. FAP-derived TnC binds the MuSC surface receptor Annexin A2. This interaction sustains MuSC self-renewal and functional integrity to support efficient muscle repair. Physiological aging reduces TnC expression, which critically impairs the regenerative capacity of aged skeletal muscle [15].

Aging also rewires FAP-immune communication. Senescent FAPs acquire a SASP-like secretome enriched in CCL2 and SPP1, which recruits macrophages and promotes M2 polarization; blockade of CCL2 or osteopontin attenuates this effect, indicating that senescent FAPs actively reshape the inflammatory niche rather than merely responding to it [50]. Complement signaling provides another age-sensitive axis. In aged injured muscle, macrophage-derived C1q activates C1r/C1s-expressing FAPs, enhances Wnt signaling, and drives collagen gene expression and fibrogenic differentiation; pharmacological complement inhibition or FAP-specific C1s ablation reduces fibrosis [60]. Importantly, elderly human muscle studies also identify FAP-macrophage co-localization and C3-mediated communication during regeneration, suggesting that complement-dependent FAP-immune crosstalk is not limited to mouse models [90].

These changes extend beyond fibrosis. Systemic aging signals can bias FAPs toward adipogenesis: aged intestinal sEV-derived miR-214-3p promotes FAP adipogenic differentiation by suppressing Wnt/β-catenin signaling, linking the gut-muscle axis to IMAT accumulation [62]. In IBM, senescent FAPs are the predominant senescent resident population and lose collagen XV, a matrix component required for myofiber integrity and neuromuscular junction formation, thereby connecting FAP senescence with structural and neuromuscular failure [91]. Thus, aged FAP dysfunction reflects a combined loss of regenerative support and gain of maladaptive paracrine activity.

Several interventions modify aging-associated FAP responses or reduce the abundance of senescent FAPs. Resistance training reduces senescent FAP accumulation, suppresses SASP, limits M2 polarization, and prevents age-associated atrophy in rats [92]. In older adults, metformin pre-treatment during disuse lowers muscle collagen deposition, reduces senescence/SASP markers, and shifts FAPs away from a myofibroblast-like fate during re-ambulation [93]. Mechanotherapy similarly restores aged stromal transcriptional networks and ECM turnover after disuse [77], while senolytic fisetin removes senescent FAPs and rescues MPC function in progeroid muscle [94]. Therefore, aging-induced FAP dysfunction should be viewed neither as an irreversible intrinsic defect nor as a purely secondary consequence of niche decline, but as a context-dependent and therapeutically modifiable state. Current evidence remains strongest in mouse and in vitro models, with emerging but still limited human validation; conclusions should therefore be restricted to skeletal muscle and not directly extrapolated to FAP-like populations in other tissues.

### 5.3. Pathogenic FAP States in Muscular Dystrophy

Fibrotic and fatty replacement in muscular dystrophy arises from disease-stage- and tissue-specific pathogenic FAP states rather than uniform expansion. Single-cell analysis of human DMD muscle resolves FAPs into proliferative, regulatory, inflammatory, and myofibroblast-like populations enriched for CHI3L1, EFEMP1, MFAP5, TGFBR2, ECM organization, and collagen-remodeling programs [36]. In Becker muscular dystrophy, single-nucleus profiling identifies FAPs as the most transcriptionally altered non-myogenic population, with expansion of LUM+/DCN+/COL1A1+ profibrogenic activated FAPs [95]. Spatial transcriptomics further localizes FAP-enriched gene programs to active fibrotic and adipogenic lesions in DMD and BMD [96]. In D2-mdx mice, fibrotic progression correlates with enrichment of CD55+ FAP clusters and upregulation of collagen, TGF-β, WNT, NOTCH, ANGPTL, THY1, and TWEAK signaling [51].

Mechanistically, dystrophic FAPs integrate inflammatory, mechanical, and epigenetic inputs. Injured myofibers recruit macrophages that release TGF-β, activating RUNX2-dependent fibrogenic transcription in FAPs; FAP-specific Runx2 ablation or RUNX2 inhibition reduces fibrosis in dystrophic and denervated models [11]. MuSC-derived CCL5 recruits macrophages via CCR5, amplifying macrophage TGF-β release and impairing FAP clearance [34]. Complement-WNT signaling further amplifies fibrosis: FAPs and macrophages provide distinct C1 subunits that activate WNT-dependent fibrogenic programs, and C1r/s inhibition attenuates FAP fibrogenic traits and dystrophic severity [59]. Mechanical and lipid pathways converge on FAP migration and ECM deposition through PDGF-AA-mediated RhoA/ROCK2 activation [97] and LPA-LPA1-YAP signaling [76].

The responsiveness of pathological FAP programs to intervention depends on disease stage. Early HDAC inhibition redirects dystrophic FAPs toward a regenerative state, whereas late-stage FAPs develop chromatin changes conferring partial resistance, though anti-SASP effects may persist [98]. HDAC11 deficiency limits dystrophic FAP expansion by promoting apoptosis and reducing collagen production, improving inflammation, fibrosis, and function in DMD mice [99]. FAP-derived PGE2 functions as a protective autoregulatory signal; impaired PGE2 production contributes to FAP accumulation and fibrosis, while PGE2 supplementation reduces FAP numbers, fibrosis, and improves strength [81]. Conversely, macrophage-derived SPP1 promotes PDGFRα+ stromal cells toward adipogenic states, increasing intramuscular fat and reducing diaphragmatic function [45], whereas reduced EGFR activity in D2-mdx FAPs enhances adipogenic propensity and EGF stimulation suppresses fat deposition [100].

Beyond ECM deposition, dystrophic FAPs directly impair myogenesis through their secretome. DMD-derived FAPs inhibit healthy myoblast differentiation, and C4BPA is identified as an upregulated secreted factor that reduces myotube formation and contractile function; C4BPA silencing partially restores myogenic capacity [101]. Collagen VI produced by FAPs acts as a conserved regeneration-inhibiting ECM signal across DMD, OPMD, and IBM; patient-derived FAPs induce collagen deposition, myofiber atrophy, and Pax7+ cell depletion, whereas COL6 silencing restores myogenic fusion [78]. In FSHD, FAP expansion, mitochondrial dysfunction, and alternative splicing alterations occur even in non-inflamed muscle, suggesting that FAP remodeling can proceed partly independently of overt inflammation [102]. What’s more, Forskolin (FSK), an adenylyl cyclase activator, has also shown therapeutic potential. FSK improved muscle histology in both uninjured and injured DMD muscle, reducing FAP numbers, enhancing MuSCs expansion, promoting muscle regeneration, and decreasing senescence. Additionally, FSK modulated inflammation by reducing pro-inflammatory macrophages and promoting their transition toward a reparative phenotype [103].

FAPs are thus central effector cells in muscular dystrophy, linking inflammation, ECM remodeling, adipogenesis, and regenerative failure. The critical unresolved question is not whether FAPs drive pathology but when and which FAP states remain therapeutically reversible. Current evidence derives primarily from murine models, human single-cell and spatial datasets, and in vitro patient-derived FAP systems. Spatial validation of human FAP subtypes, disease-stage-specific drug responsiveness, and clinical correlation remain essential before FAP-targeted therapies can advance to translation.

## 6. Conclusions and Future Perspectives

The evidence reviewed here supports a central principle: FAPs do not simply respond to the skeletal muscle microenvironment; they actively shape it. Early work defined FAPs as bipotent stromal precursors, whereas single-cell, spatial, and multi-omic studies now position them as environment-responsive signaling hubs that integrate TGF-β, Wnt, epigenetic, immune, and mechanical inputs to coordinate MuSC activity, immune resolution, and ECM remodeling. The shift from regenerative support to disease promotion is gradual and context dependent. In aging, declining WISP1, Tenascin-C, BMP3B, and IGF-I weakens the regenerative niche before overt fibrosis or fatty infiltration appears. In muscular dystrophy, complement-Wnt amplification, chromatin remodeling, autonomous PDGFRαsignaling, and disease-specific secretomes stabilize pathogenic FAP states, helping explain why anti-inflammatory monotherapy alone has limited durability.

These insights have immediate therapeutic implications but also clear constraints. TGF-β exemplifies the challenge. It drives fibrogenic differentiation, suppresses adipogenesis, and regulates FAP survival and abundance. TGF-β-directed interventions can also affect FAP survival and abundance. As discussed in review, SB431542 reduced both fibrosis and fatty infiltration and increased FAP apoptosis in a mouse model of massive rotator cuff tear. Thus, antifibrotic effects do not necessarily favor adipogenesis. Treatment outcomes should be assessed through their effects on FAP differentiation, survival, and tissue repair in the relevant context. GSK3 inhibition suppresses FAP adipogenesis across mouse, bovine, and human systems, but excessive Wnt/β-catenin activation may affect MuSC self-renewal and other Wnt-sensitive tissues. HDAC inhibition can redirect early dystrophic FAPs toward regenerative states, whereas chronic disease may develop partial epigenetic resistance. Biomimetic ECM engineering and mechanotherapy can improve FAP paracrine function, but whether mechanical correction can overcome persistent biochemical fibrogenic cues in vivo remains unresolved. These limitations argue for stage-specific, combinatorial strategies that amplify pro-regenerative FAP functions during early repair while selectively suppressing pathogenic FAP states in chronic disease.

Future progress depends on three priorities. First, human spatial validation catch up with murine mechanistic work, particularly across disease stages, muscle groups, and fibrotic or adipogenic lesion areas. Second, FAP subpopulation heterogeneity should be linked to function through perturbation, lineage tracing, and spatial assays rather than described only by marker expression. Third, cross-species conservation of FAP regulatory modules should be tested systematically, because TGF-β responsiveness, Wnt ligand repertoires, adipogenic thresholds, and immune-stromal interactions differ across mice, cattle, pigs, and humans. By integrating these molecular, mechanical, single-cell, and translational dimensions, FAP biology can move from descriptive niche mapping toward rational therapeutic reprogramming of fibrofatty muscle degeneration.

## Figures and Tables

**Table 1:** Cross-species comparison of FAP identity, heterogeneity and functional validation. (**A**) Mouse–human comparison. (**B**) Livestock and disease-associated examples.

(**A**)				
Feature	Mouse	Human	Conservation	Evidence and Limits
Positive isolation markers	Sca-1 and/or PDGFRα with lineage exclusion [19,20,21].	PDGFRα; CD201 as a validated alternative. Other validated panels use CD34 or fresh-tissue CD73 [22,23,24].	Conserved: PDGFRα-associated identity. Species-specific: murine Sca-1 strategy.	Validated isolation. CD34, CD201 and CD73 are alternative panel components, not a mandatory all-positive gate.
Negative gates and preparation	Viable singlets; CD45^−^CD31^−^; α7-integrin^−^ to exclude myogenic cells [19,20].	Viable singlets; CD45^−^CD31^−^; CD56^−^ in CD34-based panels [24]. CD73 specificity depends on fresh versus cultured cells [22,23].	Partially conserved: shared lineage exclusion, species-adapted myogenic gates.	Protein-level validation. Digestion, culture and gating alter recovery; frequencies from different protocols are not directly comparable.
Anatomical localization	Interstitial/endomysial FAPs, distinct from the satellite-cell niche [21].	Interstitial, often vascular-associated CD201^+^ cells, distinct from sublaminar satellite cells [24].	Conserved: broad interstitial localization. Precise subset niches: partially conserved.	Tissue localization supports identity. Vascular proximity does not establish pericyte identity; ECM RNA signatures alone do not locate individual cells.
Differentiation potential	Clonal fibro-adipogenic differentiation; adipocyte formation in permissive transplantation settings [19].	Adipogenic and fibrogenic responses in purified populations under appropriate culture conditions [22,23,24]	Conserved at population level; uniform single-cell bipotency is not implied.	In vitro validation in both species; conditional transplantation evidence includes human CD201^+^ cells [24]. Neither establishes an unrestricted endogenous fate.
Adipogenic restriction by subset	MME^+^ versus MME^−^ adipogenic difference apparent under minimal induction [25].	MME^+^ more adipogenic; few MME^−^ cells differentiate even with full induction [25].	Partially conserved: MME-associated bias; assay-dependent extent differs.	Sorted-cell assays support subset-specific restriction. These experiments do not establish a universal human–mouse ranking of total FAP adipogenic capacity.
Transcriptomic identity	LUM/FBN1-associated programs; dataset-specific TEK and collagen patterns [26].	LUM/FBN1-associated programs with PDGFRA/CD34 and ECM expression [26].	Partially conserved.	Transcriptomic correspondence. Predicted endomysial/perimysial associations and developmental relationships require independent validation.
Lineage and functional validation	Osr1 developmental tracing; FAP transplantation; selected state/function assays [19,25,27]	Purified-cell differentiation, tissue association and selected xenotransplantation experiments [22,23,24].	Evidence is unequal; this is not a species-specific biological trait.	Mouse experiments resolve some lineage and in vivo questions. Human culture/xenograft results cannot be presented as endogenous human lineage tracing.
(**B**)				
Species/Context	Identity or State	Localization/Functional Finding	Conservation	Evidence and Limits
Cattle; breed comparison [28]	PDGFRA-associated FAPs; COL4A1/CFD versus COL1A1/POSTN programs.	ECM-associated endomysial/adipogenic and perimysial/fibrogenic patterns; bovine adipogenic culture perturbations.	Partially conserved with human stromal programs.	Transcriptomics, IHC and bovine in vitro validation. Postn lineage tracing was in mice, not cattle; CFD expression is not uniform across species.
Pig; birth and growth [29,30]	PDGFRA labelled FAPs; LPL/TNMD states; COL3A1, PDE4D/PDE7B and EBF1 states [29,30].	Age/breed-related stromal programs and spatial associations; inferred differentiation trajectories.	Partially conserved at the level of ECM/adipogenic programs.	Mainly transcriptomic/spatial association. Preadipocyte perturbation [31,32] is not lineage validation of atlas-defined FAP subsets.
Camel; juvenile/adult [33]	MME, GPC6, NRAP and MYO1E-labelled FAP clusters.	Development-associated subset proportions; putative MME-associated adipogenic state.	Candidate correspondence with MME-associated states; functional conservation not yet validated.	Transcriptomic association only for the FAP subset claim; direct sorted-subset differentiation and lineage tracing not reported.
Mouse; injury and dystrophy [34,35]	Tie2/VCAM1 states; Cxcl5/Ccl7-rich activated states.	State changes with injury resolution/dystrophy; Sca-1-high cells have greater adipogenic responses in vitro.	Context-dependent; exact human subset equivalence not established.	State profiling and selected culture/perturbation evidence. VCAM1 exclusion could remove activated FAPs.
Human; DMD [36]	PDGFRα^+^CD56^−^ cells; CHI3L1/EFEMP1/MFAP5/TGFBR2-associated changes.	Altered inflammatory/ECM-related states in patient-derived cells.	Partially conserved stromal activation programs; exact mouse labels not interchangeable.	scRNA-seq after approximately one week of culture; qPCR/tissue validation of selected findings. Culture may alter state representation.
Human; T2DM [22]	CD34^+^CD45^−^CD31^−^CD56^−^ FAPs subdivided by CD90.	CD90^+^ fraction has enhanced ECM-related properties; fibro-adipogenic responses measured in vitro.	Human context validated; equivalence of a mouse CD90 subset not established here.	Sorted-cell/clonal culture and patient association; tissue co-staining does not alone resolve all endothelial contributions.
Human; ageing/fatty infiltration [25]	Ageing atlas: MME^+^ and fibroblast-like fractions. Fatty infiltration: MME^+^/MME^−^ comparison.	MME^+^ fraction decreases with ageing and in fatty-infiltrated muscle [25]; MME^+^ cells retain high adipogenic competence.	Partially conserved MME-associated adipogenic bias; abundance is context-dependent.	Ageing: multiomic association. MME differentiation: human in vitro validation. Reduced abundance does not itself prove conversion into adipocytes.
Mouse and human; CLTI [37]	VCAM1^+^ state; associated NR3C1/SFRP1 program.	Mouse sorted VCAM1^+^ cells show increased adipogenesis; associated genes enriched in human ischemic muscle.	Partially conserved transcriptional correspondence; human causality not established.	Mouse differentiation/siRNA assays; human transcriptomic association. Formal 2025 article replaces the cited preprint [38].

Abbreviations: CLTI, chronic limb-threatening ischemia; DMD, Duchenne muscular dystrophy; FACS, fluorescence-activated cell sorting; FAP, fibro-adipogenic progenitor; Osr1, Odd-skipped related 1; T2DM, type 2 diabetes mellitus.

## Data Availability

No new data were created or analyzed in this study. Data sharing is not applicable to this article.
